# Microbiomic insights into the oral microbiome’s role in type 2 diabetes mellitus: standardizing approaches for future advancements

**DOI:** 10.3389/fendo.2024.1416611

**Published:** 2024-11-29

**Authors:** Huifang Guan, Shuang Zhao, Yuanfei Tan, Xinyi Fang, Yuxin Zhang, Yanjiao Zhang, Runyu Miao, Ruiyang Yin, Yiqi Yao, Jiaxing Tian

**Affiliations:** ^1^ College of Traditional Chinese Medicine, Changchun University of Chinese Medicine, Changchun, China; ^2^ Department of Tuina, The Affiliated Hospital of Changchun University of Chinese Medicine, Changchun, China; ^3^ Institute of Metabolic Diseases, Guang’anmen Hospital, China Academy of Chinese Medical Sciences, Beijing, China; ^4^ Graduate College, Beijing University of Chinese Medicine, Beijing, China

**Keywords:** diabetes mellitus, oral microbiome, periodontitis, diabetic oral microbiology, microbial diversity

## Abstract

The burgeoning field of microbiomics has unveiled significant insights into the role of the oral microbiome in the pathophysiology of Type 2 Diabetes Mellitus (T2DM), with this review focusing on recent advancements in diabetic oral microbiology, its clinical applications, and identifying factors that may affect study interpretations. A comprehensive review across various databases, including PubMed and Google Scholar, was conducted to collate original research data published in the past five years, specifically targeting studies exploring the impact of the oral microbiome on T2DM and emphasizing research that employs microbiomic approaches in clinical patient populations. The findings delineate the intricate interplay between T2DM and oral microbiome dysbiosis, highlighting significant microbial shifts following periodontal and antidiabetic treatments, and pointing to the complexity of the relationship between oral health and systemic disease. The observed oral microbial shifts in T2DM underscore the critical need for standardized research methodologies in microbiomic studies, suggesting that by adopting a unified approach, future research can more effectively elucidate the oral microbiome’s role in T2DM. This could pave the way for innovative diagnostic and therapeutic strategies in managing T2DM and its oral health complications, thus making a pertinent overview of the work within the field.

## 1 Introduction

Type 2 Diabetes Mellitus (T2DM) is a chronic metabolic disorder characterized by hyperglycemia resulting from insulin resistance and impaired insulin secretion. It is associated with high mortality and morbidity rates globally, posing significant public health challenges (1). T2DM is linked not only to systemic complications such as cardiovascular diseases, nephropathy, and cerebrovascular accidents but also to a range of oral health complications, including periodontal disease, dental caries, and oral infections (2). Poorly controlled diabetes has been shown to increase the risk and severity of periodontal disease, which can ultimately lead to tooth loss (3). Conversely, periodontal disease in individuals with diabetes can adversely affect glycemic control, heightening the risk of diabetes-related complications such as retinopathy and nephropathy (4). This bidirectional relationship, often described as a “two-way street,” underscores the complex interplay between diabetes and periodontal disease (5). Emerging evidence suggests that periodontitis may also contribute to the incidence of new cases of T2DM and gestational diabetes (6, 7). These conditions share common risk factors, notably excessive sugar consumption and tobacco use, along with underlying infection and inflammatory pathways.

The oral microbiome plays a critical role in oral and systemic health. Changes in the composition and function of the oral microbiota have been implicated in the pathogenesis of periodontal disease and may influence systemic conditions such as T2DM (8). Advances in microbiomic technologies have enabled a deeper exploration of the oral microbial communities, revealing associations between specific microbial profiles and diabetic status. However, the precise mechanisms by which T2DM influences the oral microbiota and metabolic processes remain incompletely understood.

Despite the growing body of research utilizing microbiomic approaches to study diabetic oral disease, inconsistencies in study designs, populations, and methodologies have led to varied and sometimes conflicting results. This variability hampers a comprehensive understanding of the intricate relationship between the oral microbiome and T2DM. Addressing this knowledge gap is paramount to enhancing our understanding of the complex dynamics at play and informing more effective strategies for managing the interconnected challenges of T2DM and oral health.

This review aims to systematically evaluate current literature on the oral microbiome’s role in T2DM, focusing on studies that utilize microbiomic approaches in clinical patient populations. By identifying key microbial and metabolic indicators associated with T2DM and highlighting the importance of methodological rigor and standardization, we seek to advance our understanding of the interplay between the oral microbiome and T2DM. This could pave the way for innovative diagnostic and therapeutic strategies in managing T2DM and its oral health complications.

## 2 Methodology

We conducted a comprehensive literature review to identify studies examining the role of the oral microbiome in T2DM. The search spanned publications from January 1, 2018, to October 10, 2024, and utilized multiple databases, including PubMed, the Human Oral Microbiome Database (HOMD), and the Human Microbiome Project (HMP). This approach allowed us to gather a broad spectrum of relevant literature and foundational data pertinent to our review objectives.

The initial search was performed in PubMed using combinations of keywords such as “oral,” “saliva,” “salivary,” “microbiome,” “microbiota,” “dysbiosis,” and “diabetes.” Boolean operators were employed to refine the search and maximize the retrieval of pertinent articles. This strategy yielded a total of 717 articles. After removing duplicates, we screened the titles and abstracts of the remaining studies for relevance based on predefined inclusion and exclusion criteria.

To enhance our understanding of the oral microbiome’s role in diabetes and to inform our analysis, we consulted HOMD and HMP. The HOMD provides a curated classification and comprehensive annotation of approximately 700 prokaryote species present in the human oral cavity, including bacteria associated with both health and oral diseases such as dental caries and periodontal disease (9, 10). The database offers valuable insights into key bacterial phyla and genera, such as Actinobacteria, Bacteroidetes, Firmicutes, Proteobacteria, Fusobacterium, Haemophilus, and Streptococcus, which are central to the oral microbiome.

The HMP complements this by employing advanced techniques like 16S rRNA gene sequencing and whole-genome shotgun sequencing to characterize the microbiome across different regions of the oral cavity (11). This project has highlighted the varied abundance and distribution of bacteria such as Streptococcus and Prevotella in specific oral sites, providing crucial context for understanding the microbial landscape in health and disease, including diabetic conditions.

The inclusion criteria for selecting studies were as follows: original research articles involving human subjects with T2DM; studies employing microbiomic approaches such as 16S rRNA gene sequencing, metagenomics, proteomics, or metabolomics to analyze the oral microbiome; articles published in peer-reviewed journals within the specified date range; and studies available in English. We excluded review articles, meta-analyses, case reports, editorials, studies involving animal models or *in vitro* experiments, research not directly assessing the oral microbiome in the context of T2DM, and studies lacking sufficient methodological detail or results pertinent to our review objectives.

After applying these criteria, 45 articles were selected for full-text review. Each article was thoroughly assessed for eligibility, resulting in 45 studies being included in the final analysis. Data extraction focused on study characteristics, methodological details, and key findings related to changes in oral microbiome composition associated with T2DM. We paid particular attention to the identification of specific microbial taxa, diversity measures (alpha and beta diversity), functional analyses, and reported associations with clinical parameters such as glycemic control and periodontal status.

The foundational data provided by HOMD and HMP were instrumental in classifying and understanding the bacterial species identified in the selected studies. By integrating information from these databases, we could contextualize the microbial taxa within the broader landscape of the human oral microbiome, facilitating a deeper interpretation of their potential roles in T2DM.

This comprehensive methodology, combining literature searches in PubMed with insights from HOMD and HMP, ensured a thorough and informed approach to reviewing the current state of knowledge. It allowed us to synthesize findings across studies effectively, identify patterns and gaps, and underscore the importance of standardized methodologies in advancing our understanding of the oral microbiome’s impact on T2DM.

## 3 Contributions of the human oral microbiome database and the human microbiome project

The exploration of diabetic oral microbiology has been significantly advanced by comprehensive initiatives such as the HOMD and HMP. These resources have provided invaluable insights into the complexity and diversity of the oral microbiome, facilitating a deeper understanding of its role in health and disease.

The HOMD offers a curated classification and comprehensive annotation of approximately 700 prokaryote species present in the human oral cavity, encompassing bacteria associated with both oral health and diseases like dental caries and periodontal disease. Key bacterial phyla identified within the oral microbiome include Actinobacteria, Bacteroidetes, Firmicutes, and Proteobacteria. Genera such as Fusobacterium, Haemophilus, and Streptococcus play central roles in oral microbial communities. This extensive catalog serves as a foundational reference for researchers investigating the oral microbiota’s composition and its alterations in various conditions, including T2DM (12).

The HMP has further enriched the understanding of the human microbiome by employing advanced techniques like 16S rRNA gene sequencing and whole-genome shotgun sequencing to characterize microbial communities across different body sites, including multiple regions within the oral cavity. This comprehensive analysis has highlighted the varied abundance and distribution of bacteria such as Streptococcus and Prevotella in specific oral niches. Such insights are crucial for deciphering the microbial landscape in health and disease states, providing context for changes observed in diabetic conditions.

By leveraging the data and resources provided by HOMD and HMP, researchers can identify specific microbial taxa associated with T2DM, explore shifts in microbial diversity, and investigate potential mechanisms linking the oral microbiome to systemic metabolic processes. These databases enable the identification of non-culturable bacterial populations and facilitate comparative analyses across studies, thereby advancing the field of diabetic oral microbiology. The foundational knowledge gained from HOMD and HMP supports the development of targeted diagnostic markers and therapeutic strategies aimed at mitigating oral health complications in T2DM patients.

## 4 Differences in oral microbiota composition between T2DM patients and ND individuals

### 4.1 Analyzing oral microbiota in T2DM and ND individuals via 16S rRNA gene sequencing

The advancement of 16S rRNA-based next-generation sequencing technology is used to identify the structural and functional characteristics of nonculturable bacterial communities in the oral cavity of healthy and diseased persons (13). A study investigated periodontal bacterial differences between T2DM patients and ND controls, using 16S rDNA sequencing of subgingival plaque samples (14). T2DM individuals showed differences in bacterial composition, with elevated levels of specific phyla, such as Actinobacteria, Proteobacteria, and Bacteroidetes, in periodontitis cases (14). Significant differences were observed in genera such as Prevotella, Tannerella, Actinomyces, and Aggregatibacter between diabetic and non-diabetic (ND) groups. These findings underscore the impact of T2DM on subgingival microbial diversity and suggest that diabetes may influence the periodontal microbial environment.More recently, a matched case-control study meticulously examined the salivary microbiota in elderly Japanese patients with T2DM and compared it with that of age- and sex-matched controls, observing significant differences in the overall structure of the salivary microbiota between the two groups, particularly with an abundance of the phylum Firmicutes in the T2DM group and Bacteroidetes in the controls (15). Utilizing random forest classification, a predictive model based on the salivary microbiota demonstrated high efficiency in distinguishing T2DM patients from controls, indicating the salivary microbiome’s potential in T2DM prediction (15). These findings underscore the importance of personalized oral health management for elderly T2DM patients, paving the way for innovative diagnostics and treatments focused on microbiota characteristics.

Furthermore, A study focusing on Chinese patients with T2DM employed Illumina sequencing of the V1-V2 region of the 16S rRNA gene (16). This approach unveiled a distinct increase in the Firmicutes/Bacteroidetes ratio alongside specific genera such as Neisseria, Streptococcus, Haemophilus, and Pseudomonas that were associated with T2DM (16). Furthermore, a study from a Chinese cohort utilized 16S rDNA sequencing to compare the saliva microbial composition between T2DM patients and healthy controls (17). The findings revealed a notable decrease in the relative abundances of Fusobacteriota and Campilobacterota and an increase in Proteobacteria in the saliva of T2DM patients compared to healthy individuals (17). Moreover, Real-Time Quantitative Polymerase Chain Reaction analysis detected heightened expression of inflammatory markers such as lipopolysaccharide, apoptosis-associated speck-like protein, Caspase-1, Caspase-11, NLRP3, and interleukin -1β, alongside reduced levels of insulin receptor substrate-1 in T2DM patients (17). This research indicates that alterations in oral microbial populations may influence T2DM progression by activating the NLRP3 inflammasome pathway.

By isolating the oral microbiome’s characteristics in medicated T2DM patients, a study enriches our understanding of diabetes’ influence on oral health (18). Notably, the study found that the class Synergistia and the genus TG5, both associated with periodontitis, were more common in the control group, while no specific bacterial taxa showed significant enrichment in diabetic patients (18). Moreover, this investigation challenges prior assumptions by revealing no significant disparities in alpha and beta diversity of the oral microbiome between diabetic patients and control (18). This outcome prompts a reevaluation of the direct impact of diabetes on oral microbial diversity, proposing that medication, lifestyle, or dietary factors might play more influential roles in shaping the oral microbiome than diabetes itself (18). Similarly, another study from Korea also found no significant differences in the alpha and beta diversities of the oral mucosal microbiomes between diabetic and ND patients, indicating a subtle relationship and emphasizing the necessity for standardized methodologies in microbiome research (19).

A comprehensive investigation into the oral microbiome of South Africans with periodontal disease across glycemic statuses identified Fusobacteria and Actinobacteria as more abundant in subjects with diabetes, using 16S rDNA sequencing of multiple regions (20). This study also noted alterations in microbiota composition in relation to periodontal disease severity and glycemic status, with variations in Actinobacteria and Bacteroidetes abundance linked to gingival bleeding and diabetes (20). In a pioneering study conducted in Indonesia, researchers used MinION nanopore platform for 16S rRNA amplicon sequencing to investigate the subgingival biofilm in subjects with periodontitis, comparing those with diabetes to those without (21). The study identified six specific bacterial genera and species within the subgingival areas, noting differences in their quantities between the groups (21). Tannerella forsythia was significantly more abundant in the subgingival biofilms of the diabetic group, whereas Aggregatibacter sp. was found at lower levels. Fusobacterium sp. showed similar presence in both groups, while Veillonella sp. was more abundant in subjects with diabetes (21). This study highlights the increased diversity of periodontopathogens in diabetic subjects with periodontitis, shedding light on how systemic conditions like diabetes can influence subgingival biofilm composition.

In a pioneering study, researchers employed a multi-omics approach, integrating proteomics, lipidomics, metabolomics, and 16S DNA sequencing, to examine the dental plaque from patients suffering from diabetes and periodontal disease (22). This comprehensive analysis unveiled a unique microbial dysbiosis in these patients, characterized by specific microbial communities and the elevation of host-specific proteins and associated lipids in plaques from individuals with periodontal disease. Notably, the study discovered that the oral community member Lautropia mirabilis synthesizes monomethyl phosphatidylethanolamine, a lipid rarely found in the oral microbiota (22). Transitioning to a related avenue of inquiry, another investigation utilized 16S rRNA gene sequencing alongside Ultra High-Performance Liquid Chromatography-Mass Spectrometry based metabolomics to study the subgingival microbiome and its metabolic output in periodontitis patients with T2DM (DAP) and T2DM patients without periodontitis. By categorizing participants into distinct groups, this study shed light on the subtle microbial and metabolic shifts that accompany T2DM and its impact on periodontal health (23). The results elucidated less marked changes in the subgingival microbiota associated with health-to-disease progression in T2DM subjects, despite similar clinical presentations. Key bacteria identified included the Eubacterium nodatum group and Filifactor, alongside metabolic pathways such as butyrate and phenylalanine metabolism, suggesting their significant role in the pathogenesis of periodontitis within T2DM contexts (23). Moreover, this study underscored the intertwined relationship between subgingival microorganisms, metabolic dysregulation, blood glucose levels, and T lymphocyte immunity, offering novel insights into the complex dynamics governing microbial presence and systemic responses in DAP.

In conclusion, these studies collectively highlight the intricate and multifaceted relationship between changes in the oral microbiome and T2DM along with its complications. Beginning with the application of 16S rRNA sequencing to explore microbial compositions, research has revealed significant differences between T2DM patients and ND individuals, and associations with inflammatory responses and metabolic pathways. However, the direct impact of diabetes on microbial diversity remains uncertain, with other factors like medication, lifestyle, or diet potentially playing influential roles. The presence of periodontal disease further complicates the interaction between systemic health and the oral microbiome. Multi-omics approaches have deepened our understanding of how microbial dysbiosis relates to host metabolism and immune responses. These findings underscore the complexity of the relationship and highlight the urgent need for standardized methodologies in microbiome research to accurately elucidate the role of the oral microbiome in T2DM.

### 4.2 Analyzing oral microbiota in T2DM and ND Individuals via whole metagenomic shotgun sequencing

The research on the Whole metagenomic shotgun sequencing method will further deepen our understanding of these complex relationships, especially the differences at the microbial classification level, providing us with a new perspective to explore the connections between these two conditions. In a study conducted in Italy, Fusobacterium nucleatum subsp. nucleatum was found in higher abundance in diabetics (24). Additionally, Rothia dentocariosa, associated with a healthy periodontium, showed higher levels in ND individuals without periodontitis, potentially offering a protective effect (24). Furthermore, metabolic pathways related to Lipopolysaccharide biosynthesis, nucleotide metabolism imbalance, and ferroptosis were significantly more prevalent in patients with both periodontitis and diabetes, suggesting a bidirec tional Chronic Periodontitis (CP) link between these conditions, especially from diabetes to periodontitis (24). Similarly,in a pivotal study conducted in Uttar Pradesh, India, researchers undertook a non-randomized observational trial to dissect the differences in salivary microflora between diabetic and ND individuals through metagenomic analysis (25). Phylum Bacteroidetes and Fusobacteria were notably abundant in diabetics, contrasting with the higher presence of Proteobacteria in NDs (25). Meanwhile, no significant difference was observed in the abundance of Actinobacteria and Firmicutes between the two groups. Among the genera identified, Veillonella, Prevotella, Porphyromonas, Leptotrichia, Lactobacillus, and Streptococcus were prevalent in diabetic individuals, whereas Capnocytophaga and Neisseria were more abundant in NDs (25). A study from the University of California found that Type 2 diabetes and periodontitis are linked to reduced richness and diversity in the subgingival microbiome (26). However, T2DM presence alone did not significantly impact the relative abundance of specific species associated with periodontitis (26). The microbiome transition from a healthy state in periodontitis was less pronounced in T2DM patients than in ND individuals, despite similar clinical symptoms. Additionally, longitudinal analysis showed T2DM patients were more prone to dysbiosis, likely due to compromised metabolism and immune function (26). Extending these findings, one study highlight the profound modulatory capacity of dietary constituents—including but not limited to the consumption of sugary snacks and marine-derived proteins—on the composition of the oral microbiome (27). A salient divergence in this study’s approach is its rigorous examination of the Mediterranean diet’s ramifications, positing a correlation with a diminished prevalence of obesity and T2DM alongside potentially salutary modifications within the oral microbial milieu (27). Utilizing a nested case-control design complemented by the application of the Mediterranean Diet Adherence Screener index to gauge MedDiet fidelity, the research furnishes pivotal insights into the diet’s potential to recalibrate the oral microbiome, thereby heralding dietary intervention as a strategic avenue to ameliorate diabetes-induced dysbiosis within the oral microbial community.

Furthermore, the integration of shotgun sequencing with genome-resolved metagenomics offered deeper insights into bacterial content variations, underscoring the specialized microbial signatures associated with CP when juxtaposed with T2DM (28). The study involved 15 patients with CP, 15 with both CP and T2DM (CPT2DM), and 16 healthy controls, with an average age over 53 and an equitable gender distribution. Overweight and obesity were more common in the CPT2DM group, though clinical indices between CP and CPT2DM groups were similar.Metagenomic analysis showed higher alpha diversity in the CPT2DM group compared to CP and controls. Biomarker analysis revealed elevated Porphyromonadaceae in periodontitis cases. In the CPT2DM group specifically, Streptococcaceae and Pasteurellaceae were lower, while Leptotrichiaceae and Neisseriaceae were higher, with Veillonellaceae reduced. These findings highlight unique microbial interactions in the presence of T2DM.

**Table 1** shows that some of these studies demonstrate opposite findings. Most studies have found significant differences in the oral microbiota between individuals with T2DM and those without, pointing to specific microbial markers that could be instrumental in understanding and managing the disease. These differences, ranging from microbial diversity to the presence of unique bacterial taxa, underscore the potential of microbiome analysis in refining diagnostic and therapeutic approaches for T2DM. Conversely, A small number of studies contends that no stark differences exist in the oral microbiome composition between diabetic and ND individuals. This perspective suggests a more nuanced interplay between oral health and diabetes, emphasizing the need for standardized methodologies in microbiome research to reconcile these divergent findings. The juxtaposition of these studies highlights the complexity of the oral microbiome’s relationship with systemic health and calls for further investigation to elucidate the true nature of these interactions.

**Table 1:** Clinical studies on microbiome composition differences in T2DM and non-T2DM populations.

No	Country	Studies	method	Outcomes	References
1	Bangladesh	*N* = 24: (i)T2DM group, (ii)ND group	16S rRNA gene sequencing	Diabetic plaques showed a distinctive bacterial composition with an increased prevalence of gram-positive bacteria, including specific Streptococcus and Staphylococcus species (↑).	(14)
2	Japan	*N* = 84: (i)T2DM group, (ii)ND group	16S rRNA gene sequencing	The phylum *Firmicutes* was abundant in patients with T2DM(↑), whereas the phylum *Bacteroidetes* was abundant in controls(↑).	(15)
3	China	*N*=442: (i)T2DM groups, (ii) healthy controls	16S rRNA gene sequencing	The Firmicutes/Bacteroidetes ratio increased in T2DM and T2DM patients presented significantly higher numbers of Neisseria, Streptococcus, Haemophilus, and Pseudomonas genera(↑), and lower numbers of Acinetobacteria compared(↓).	(16)
4	China	*N*=34: (i)T2DM, (ii) healthy controls	16S rRNA gene sequencing	The relative abundance of Proteobacteria in patients with T2DM was higher than that of healthy people(↑), whereas the relative abundances of Fusobacteriota and Campilobacterota in the saliva of patients with T2DM were lower than those of healthy people(↓).	(17)
5	Portugal	*N* = 46: (i) T2DM, (ii) healthy controls	16S rRNA gene sequencing	There were no significant differences in the oral microbiome profiles in diabetic and ND patients (-).There was no significant difference in alpha and beta diversity levels in diabetic and ND groups (-).	(18)
6	Korea	*n* = 26: (i)T2DM group, (ii)ND group	16S rRNA gene sequencing	There was no significant difference in alpha and beta diversity levels in diabetic and ND groups(-).	(19)
7	South African	*N*=128: (i)normotolerant, (ii) prediabetes, (iii)screen-detected T2DM, (iv) T2DM receiving treatment	16S rRNA gene sequencing	Fusobacteria and Actinobacteria were significantly more abundant in subjects with diabetes (↑), while Proteobacteria were less abundant(↓).	(20)
8	Indonesia	*N*=12: (i)T2DM+ periodontitis, (ii) non-T2DM+ periodontitis groups	16S rRNA gene sequencing	Tannerella forsythia was the most abundant in the diabetes group’s subgingival biofilms among red complex bacteria(↑). Aggregatibacter sp. (Proteobacteria) was at lower levels(↓), whereas Fusobacterium sp. (Orange complex) was similar in both groups(-). Veillonella sp. was abundant in the diabetes group(↑).	(21)
9	USA	*N=97:* (i) pre-T2DM/T2DM, (ii) pre-T2DM/T2DM and periodontal disease, (iii) periodontal disease, (iv) healthy subjects	16S rRNA gene sequencing, proteomics, lipidomics, and metabolomics	Plaque samples from Pre-DM/DM patients contained higher abundances of Fusobacterium and Tannerella than plaques from metabolically healthy patients (↑).	(22)
10	USA	*N*= 41: (i) T2DM with/without periodontitis, (ii) periodontitis, (iii) healthy subjects	16S rRNA gene sequencing, metabolomics, flow cytometry	The differential bacteria between T2DM and ND subjects were Haemophilus and Flexilinea(↑).	(23)
11	Italy	*N*=12: (i) moderate to severe periodontitis and T2DM, (ii) patients affected by moderate tosevere periodontitis but no T2DM, (iii) patients affected by T2DM but no periodontitis, and (iv) healthy subjects.	Whole metagenomic shotgun sequencing, MEGAN6 and MetaPhlAn 3.0 analysis	Fusobacterium nucleatum subsp. vincentii and F. nucleatum subsp. nucleatum were more abundant in ND with periodontitis, while F. nucleatum subsp. nucleatum showed a higher abundance in diabetics overall (↑). Rothia dentocariosa, associated with a healthy periodontium, was enriched in ND without periodontitis, potentially serving a protective role (↑).	(24)
12	India	*N*=136: (i)T2DM, (ii) non-T2DM groups	Whole metagenomic shotgun sequencing	The genera Veillonella, Prevotella, Porphyromonas, Leptotrichia, Lactobacillus, and Streptococcus were comparatively over the odds with the diabetics (↑).	(25)
13	USA	*N*=31: (i)T2DM groups, (ii) non-T2DM groups	Whole metagenomic shotgun sequencing	No significant difference (-).	(26)
14	Spain	*N*=121: (i)T2DM groups, (ii) non-T2DM groups	Whole metagenomic shotgun sequencing	Richness and diversity of the salivary microbiome were reduced in participants with T2DM compared to those without diabetes (↓).	(27)
15	Russia	*N*=15: (i) CP groups, (ii) CPT2DM, (iii)healthy subjects.	16S rRNA gene sequencing +Whole metagenomic shotgun sequencing	The alpha diversity in the CPT2DM group was increased compared to the CP and Control groups(↑). The CPT2DM group showed a lower relative abundance of Streptococcaceae and Pasteurellaceae (↓) and a higher abundance of Leptotrichiaceae (↑) compared to the CP and Control groups. The relative abundance of Veillonellaceae was lower in the CPT2DM group compared to the CP group (↓), while Neisseriaceae was increased in the CPT2DM group relative to the CP group (↑).	(28)

↑ Indicates an increase in number or abundance, and ↓ indicates a decrease in the number of oral bacteria, -indicates similarity in the number of oral bacteria.

### 4.3 Microbiome interaction network for T2DM, non-T2DM, and periodontitis groups

To visually represent the complex interactions among the oral microbiota across T2DM, non-T2DM, and periodontitis groups, we constructed a Microbiome Interaction Network based on data compiled in **Table 1**. This network graph highlights the relative abundances and connections of specific bacterial taxa across these groups, facilitating a comparative analysis that underscores unique and shared microbiome signatures associated with T2DM and periodontitis.

#### 4.3.1 Construction methodology

This network was derived from the microbial composition data in **Table 1**, synthesizing findings from multiple studies that assessed the oral microbiome using 16S rRNA gene sequencing, metagenomics, and other omics approaches. We selected key microbial taxa identified as significantly abundant or depleted in at least one of the groups (T2DM, non-T2DM, and periodontitis). Each microbial taxon is represented as a node, with node color distinguishing taxa by study-reported prevalence in T2DM (red), non-T2DM (blue), or overlapping groups (purple for common taxa between T2DM and periodontitis). Nodes are linked by edges that indicate observed interactions or co-occurrence patterns derived from reported associations in the literature. The color intensity and edge thickness reflect the strength of these associations, providing a nuanced view of how these microbial communities differ in health and disease.

#### 4.3.2 Results interpretation

The network graph reveals that several taxa exhibit higher abundance in T2DM patients, such as Fusobacterium, Streptococcus, and Prevotella, suggesting these as potential microbial markers of diabetic oral dysbiosis. Tannerella forsythia and Aggregatibacter were similarly prominent in periodontitis cases with T2DM, whereas Veillonella showed significant enrichment in T2DM regardless of periodontal status. Conversely, taxa like Neisseria and Rothia were more prevalent in non-diabetic, periodontitis-free controls, supporting their potential role in maintaining oral health. Additionally, the co-occurrence of Fusobacterium nucleatum subspecies among diabetic and periodontitis groups suggests its contribution to the shared pathogenic pathways, which could exacerbate the progression of both T2DM and periodontal disease.

#### 4.3.3 Relevance and implications

This microbiome interaction network illustrates the intricate relationships within the oral microbial ecosystem and offers insights into how systemic conditions like T2DM may shape or be shaped by the microbial environment. By visualizing these interconnections, the network highlights potential diagnostic markers and therapeutic targets in the oral microbiome. This graphical representation supports the need for standardized approaches in microbiome research, as varied methodologies could influence our understanding of these microbial interactions and their impact on systemic health outcomes.

## 5 Exploring microbiomic insights into oral microbiota among pre-diabetes and T2DM patients

### 5.1 Comparative analysis of microbiomic profiles in pre-diabetes and T2DM patients

Recent studies have increasingly focused on examining how variations in glycemic status affect the composition and diversity of the oral microbiome, highlighting consistent patterns of microbial dysbiosis linked to worsening metabolic conditions (**Table 2**). Across these studies, a common theme emerges: elevated blood glucose levels correlate with notable shifts in oral microbiota composition, often marked by reduced microbial diversity and an enrichment of pathogenic taxa. For instance, a study in China categorized elderly individuals by fasting glucose levels and found that higher blood glucose levels were associated with distinct oral microbiome dysbiosis, particularly with the enrichment of bacterial genera such as Leptotrichia, Staphylococcus, Catonella, and Bulleidia in the very high glucose group (29). These findings suggest that oral microbial imbalances may not only reflect metabolic disturbances but could also exacerbate hyperglycemia-related diseases (29). Similarly, a study from Qatar examined the oral microbiota of obese hyperglycemic individuals compared to lean controls. While overall diversity between the two groups showed no significant differences, a higher Firmicutes/Bacteroidetes ratio—an obesogenic microbiome signature—was observed in obese individuals, further linking metabolic status with shifts in microbial composition (30). Building on this, a Brazilian study investigated the impact of glycemic control on the subgingival microbiome in CPT2DM. The study found that patients with better glycemic control (HbA1c < 7.8%) exhibited higher microbial diversity, while those with poorer control (HbA1c ≥ 8%) had a higher prevalence of fermenting species associated with propionate and succinate production. Inadequate glycemic control was also associated with elevated levels of Streptococcus anginosus and Streptococcus agalactiae, potentially indicating subgingival sites as reservoirs for invasive pathogens (12). These findings reinforce the concept that glycemic status plays a pivotal role in modulating the oral microbiome, with significant implications for both metabolic and periodontal health.

**Table 2:** Clinical studies on oral microbiota composition in pre-diabetes and T2DM patients.

No	Country	Studies	Methods	Outcomes	References
1	China	*N*=150: according to their fasting glucose level: (i) normal, (ii)high, (iii)very high	16S rRNA gene sequencing	Very high group showed deterioration of the metabolic phenotype and dysbiosis of the oral microbiota, including Leptotrichia, Staphylococcus, Catonella,andBulleidia were significantly enriched in the VH group(↑).	(29)
2	Qatar	*N=73:* (i) obese,(ii)and lean control	16S rRNA gene sequencing	Obese hyperglycemic individuals exhibit a higher Firmicutes/Bacteroidetes ratio in their oral microbiome(↑).	(30)
3	Brazil	N=21 T2DM non-smoking patients with CP allocated into two groups based on glycaemic status: (i) inadequate glycaemic control (HbA1c ≥ 8%), (ii) adequate glycaemic control (HbA1c < 7.8%)	16S rRNA gene sequencing (V5-V6 regions)	Patients with adequate glycaemic control exhibited higher microbial diversity in the subgingival biofilm (↑). Inadequate glycaemic control favored fermenting species associated with propionate/succinate production(↑), while butyrate/pyruvate producers decreased (↓). Higher abundances of Streptococcus anginosus group and Streptococcus agalactiae were observed in the inadequate control group, indicating potential reservoirs of invasive pathogens(↑).	(12)
4	Netherlands	*N*=20: (i) pre-T2DM, (ii)T2DM	16S rRNA gene sequencing	Statistically significant associations between oral anterior α-diversity and peptide YY, and posterior α-diversity and polypeptide(↑).	(31)
5	Brazil	*N=*48: (i)T2DM with periodontitis,(ii) healthy subjects.	16S rRNA gene sequencing	Enrichment of sulphate-reducers and depletion of butyrate-producers(↑).	(32)
6	China	*N=*257: (i)T2DM, (ii) healthy subjects.	16S rRNA gene sequencing	The oral marker bacteria of T2DM were found, such as Actinobacteria, Streptococcus, Rothia(↑).	(33)
7	China	N=102: (i) nondiabetic people, (ii) treatment-naïve T2DM patients, (iii) T2DM patients with metformin treatment, (iv)T2DM patients with combined medication treatment (insulin plus metformin or other hypoglycemic drugs)	16S rRNA gene sequencing	In nondiabetic individuals, Proteobacteria was the most abundant phylum (>40%), significantly reduced in treatment-naïve T2DM patients (↓). Bacteroidetes, Firmicutes, and Fusobacteria were increased in T2DM (↑). Prevotella was most abundant in T2DM(↑) while Neisseria and Haemophilus dominated in non-T2DM (↑). Significant species changes in T2DM included Prevotella_aurantiaca (↑), Prevotella_oris(↑), Streptococcus_mutans (↑), and Pseudomonas_beteli (↓).	(34)
8	Israel	N=200: (i) personalized postprandial glucosetargeting diet, (ii) the standard of care Mediterranean diet.	whole-genome shotgun sequencing	In the oral microbiome, of the broad measurements of richness, diversity and human cell shedding, only diversity had a significant increase in the PPT group (↑)and no significant changes were observed in the MED group(-).	(35)
9	China	*N*=22: (i) CPT2DM, (ii) non- CPT2DM.	16S rRNA gene sequencing	T2DM subjects had a higher proportion of total clones of Porphyromonas, Fretibacterium, Fusobacterium, Rothia, and Filifactor, as well as Eubacterium saphenum in subgingival plaque(↑), but a lower proportion of total clones of Comamonadaceae and Comamonas in subgingival plaque and a lower proportion of Actinomyces, Alloprevotella, Oribacterium, Campylobacter, Lautropia in saliva(↓).	(36)
10	China	*N*=31: (i)treatment-naïve patients with T2DM received sole treatment with Cana (100 mg/day), (ii)10 healthy controls	16S rRNA gene sequencing	After Cana treatment, a significant increase of Prevotella and Veillonella(↑), both to be closely associated with short-chain fatty acid.	(37)
11	China	N = 133: (i)healthy individuals, (ii)periodontitis patients, (iii)T2DM patients, (iv)periodontitis patients with T2DM (v) DAP patients treated with Metformin.	16S rRNA gene sequencing	The Periodontitis and Diabetes and Periodontitis groups showed a significantly higher diversity of saliva microbiota(↑), while the T2DM and Metformin groups had no significant difference in microbial abundance (-) but showed a trend of increasing diversity (↑).	(38)

↑ Indicates an increase in number or abundance, and ↓ indicates a decrease in the number of oral bacteria, -indicates similarity in the number of oral bacteria.

Despite the overall microbiome diversity remaining similar across groups, this ratio’s elevation in obese individuals suggests a potential microbial marker linked with hyperglycemic conditions. These findings underline the intricate relationship between microbiome composition and metabolic health states, highlighting the necessity of further research to unravel the complexities of oral microbiota in the context of obesity and pre-diabetes. In tandem with these investigations, another study delved into the specific impacts of T2DM on the oral microbiome’s α-diversity and its subsequent effects on entero-endocrine markers (31). It was found that a heightened oral posterior α-diversity could potentially influence the glucose-dependent insulinotropic polypeptide response in individuals with T2DM (31). This research adds another layer to our comprehension of the oral microbiome’s influence on metabolic pathways, indicating the potential for targeted microbial interventions in managing T2DM-related metabolic issues (31).

Building on the nuanced exploration of the oral microbiome’s role in T2DM, recent studies have shifted focus towards the intricate interplay between systemic conditions and local oral environments. One such investigation unveils the pronounced effect of hyperglycemia on the oral microbiome’s composition, spotlighting the emergence of specific bacterial taxa like Treponema and Desulfobulbus in response to systemic metabolic shifts (32). This study’s novelty lies in its detailed analysis of salivary pH’s impact on microbial dynamics, underlining the significant influence of local environmental factors, such as pH, on the diabetic oral microbiome (32). The discovery of Desulfobulbus as a notably enriched organism in T2DM subjects, and its negative correlation with butyrate-producing bacteria, further underscores the complex relationship between systemic health and oral microbial composition, suggesting that salivary conditions and systemic hyperglycemia jointly mold the oral microbial ecosystem (32).Transitioning from this understanding, another pivotal study delves into the characterization of oral microbiota in T2DM patients, pinpointing key oral marker organisms like Actinobacillus and Streptococcus (33). Expanding the scope of inquiry, this research bridges the gap between oral and intestinal microbiomes, presenting an innovative perspective on the possible ectopic colonization of oral microbiota in the gut (33). This exploration not only sheds light on the mutualistic relationship between oral and intestinal microbiomes but also paves the way for understanding how disruptions in this balance contribute to the systemic impact of T2DM.

Subsequently, we explore a series of studies that assess the effects of periodontal treatment, dietary intervention and antidiabetic medication on the oral microbiome, providing a comprehensive view of how therapeutic interventions can modify microbial composition and activity in the context of diabetes and oral health. In recent research, the salivary microbiome of nondiabetic individuals, treatment-naïve diabetic patients, and those treated with metformin or combined insulin therapy were compared, highlighting significant microbial diversity changes linked to the onset and treatment of T2DM. Notably, specific bacterial taxa, including Blautia_wexlerae, Lactobacillus_fermentum, Nocardia_coeliaca, and Selenomonas_artemidis, were identified as fluctuating throughout diabetes progression and treatment. These differential bacteria correlated with treatment types and pathological changes, suggesting their potential as biomarkers for distinguishing diabetic states and evaluating treatment efficacy (34). Similarly, researchers uniquely analyzed the oral and gut microbiomes before and after a dietary intervention to understand their response to diet and T2DM (35). The study found that, contrary to expectations, the oral microbiome showed greater genetic strain dynamics compared to the gut microbiome, despite the gut experiencing more pronounced compositional changes. A detailed strain replacement analysis revealed this higher genetic variability in the oral microbiome, challenging assumptions about the stability of microbial communities in the oral cavity versus the gut (35). The findings suggest that the oral microbiome’s genetic composition can significantly fluctuate, potentially influenced by dietary factors and the host’s metabolic status. Specifically, the oral microbiome of individuals with T2DM or obesity showed marked genetic changes, especially in species like Actinomyces naeslundii, Fusobacterium nucleatum, and Leptotrichia buccalis, known for their associations with oral health and metabolic diseases. This genetic dynamism within the oral microbiome underscores its sensitivity to external factors such as diet and provides insights into its role in mediating the effects of dietary interventions on metabolic and immune health. Exploring the dynamic interplay between periodontal health and T2DM, the first study conducted at the Second Affiliated Hospital of Chongqing Medical University provides invaluable insights into how nonsurgical periodontal therapy influences the oral microbiota and clinical outcomes in patients with and without T2DM (36). This investigation underscores the significant shifts in microbial diversity and composition post-treatment, highlighting the therapy’s potential to enhance periodontal health and glycemic control. Building upon these findings, a subsequent study delves further into the impact of specific treatments on the oral microbiome, focusing on Cana treatment (37). Through 16S rRNA sequencing, this research identifies a marked increase in Prevotella and Veillonellfa populations post-treatment—microbes intimately linked to short-chain fatty acids production. In addition, recent a study, the impact of T2DM and hypoglycemic therapy on the salivary microbiome in periodontitis patients was examined to identify potential biomarkers for early T2DM detection (38). Saliva samples from healthy individuals, periodontitis patients, T2DM patients, DAP, and those treated with Metformin were analyzed using 16S rRNA gene sequencing. Findings revealed a higher diversity of saliva microbiota in periodontitis patients with or without T2DM compared to healthy individuals, with no significant microbial abundance difference in T2DM and Metformin groups but an increasing diversity trend (38). Notably, Prevotella copri, Alloprevotella rava, and Ralstonia pickettii proportions increased in periodontitis patients, decreasing after glycemic control. The study suggests hypoglycemic therapy can modify salivary microbiota, potentially aiding in managing periodontitis in diabetic patients. This observation not only complements the previous study’s conclusions on therapy-induced microbial shifts but also offers a more nuanced understanding of how treatments may modulate the oral microbiota, potentially influencing broader metabolic health markers and periodontal disease management strategies in T2DM patients. Extending the exploration of therapeutic interventions and their impact on the oral microbiome in T2DM patients, a randomized controlled trial evaluated the efficacy of scaling and root planing with adjunctive Ndlaser therapy compared to SRP alone in DAP (39). The study found that combining Ndlaser therapy with SRP led to greater improvements in periodontal parameters and significant reductions in fasting plasma glucose levels compared to SRP alone. Microbiome analysis revealed a shift from disease-associated taxa, such as Porphyromonas, toward health-associated taxa, including Fusobacteria and Leptotrichia, following the adjunctive laser treatment. These findings suggest that integrating Ndlaser therapy with conventional periodontal treatment not only enhances periodontal healing but also improves glycemic control in T2DM patients, potentially by promoting a beneficial shift in the subgingival microbiome composition.

### 5.2 Interaction network of oral microbiota in T2DM, Non-T2DM, and periodontitis groups

**Figure 1:**
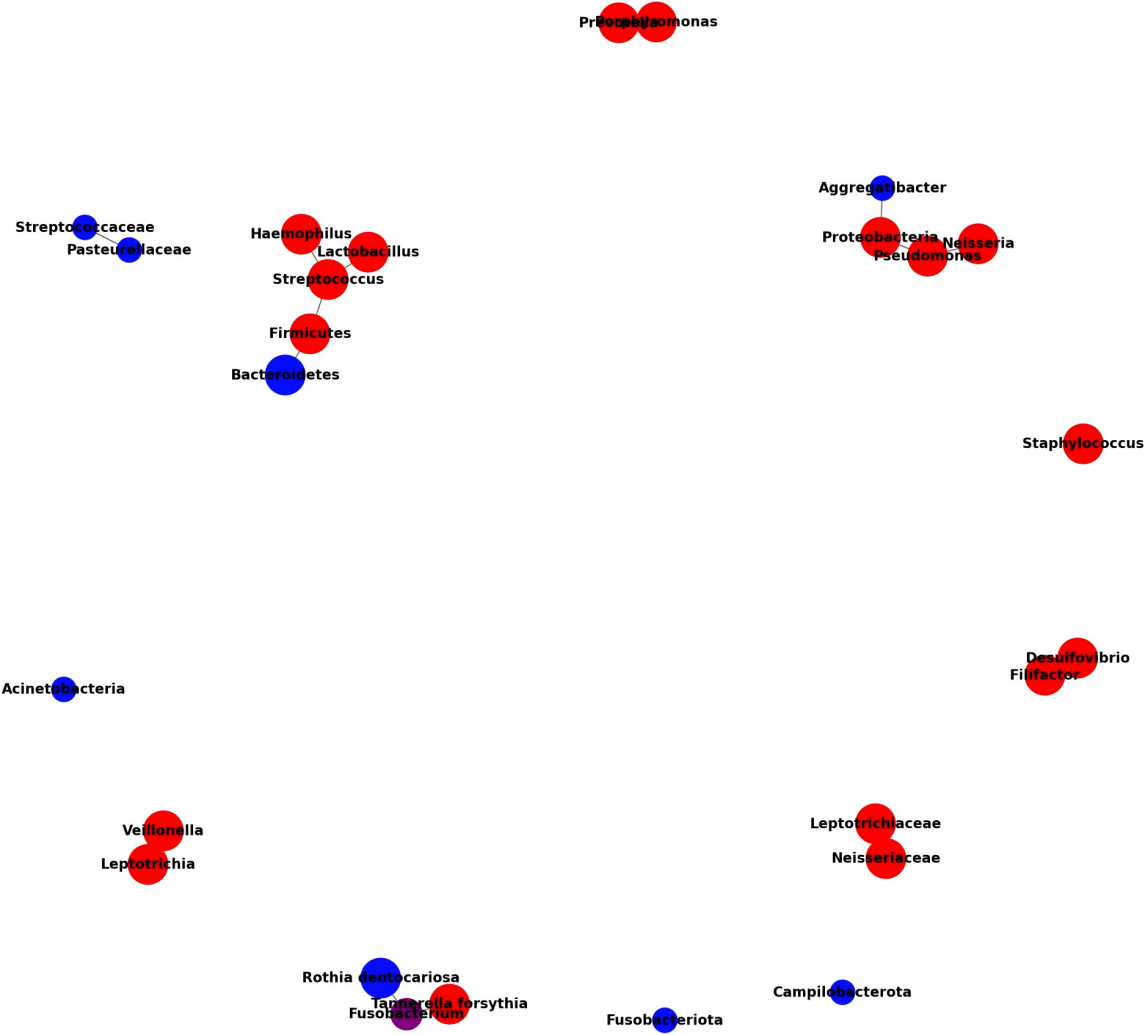
Microbiome interaction network across T2DM, Non-T2DM (ND), and periodontitis groups. This network illustrates the microbial composition and relationships across different health conditions, focusing on T2DM, non-T2DM, and periodontitis associations. Node colors indicate the primary group associations of each taxon: red for bacteria predominantly found in T2DM patients, blue for those mainly in ND individuals, and purple for taxa commonly found in both groups. Node size represents the relative abundance of each taxon within its associated group(s), with larger nodes signifying an increased presence in the given group. Edges (connecting lines) represent documented interactions or co-occurrences among bacteria, highlighting taxa that commonly coexist or exhibit functional relationships. Key patterns include an increased presence of T2DM-associated bacteria like Desulfovibrio and Haemophilus, a decrease in ND-specific taxa like Streptococcaceae and Pasteurellaceae, and shared taxa like Streptococcus, which appear across multiple groups. This network underscores the distinctive microbial profiles associated with each health condition and suggests potential microbial interactions that may influence oral and systemic health in T2DM contexts. Microbiome interaction network for T2DM, Non-T2DM, and periodontitis groups.

**Figure 2:**
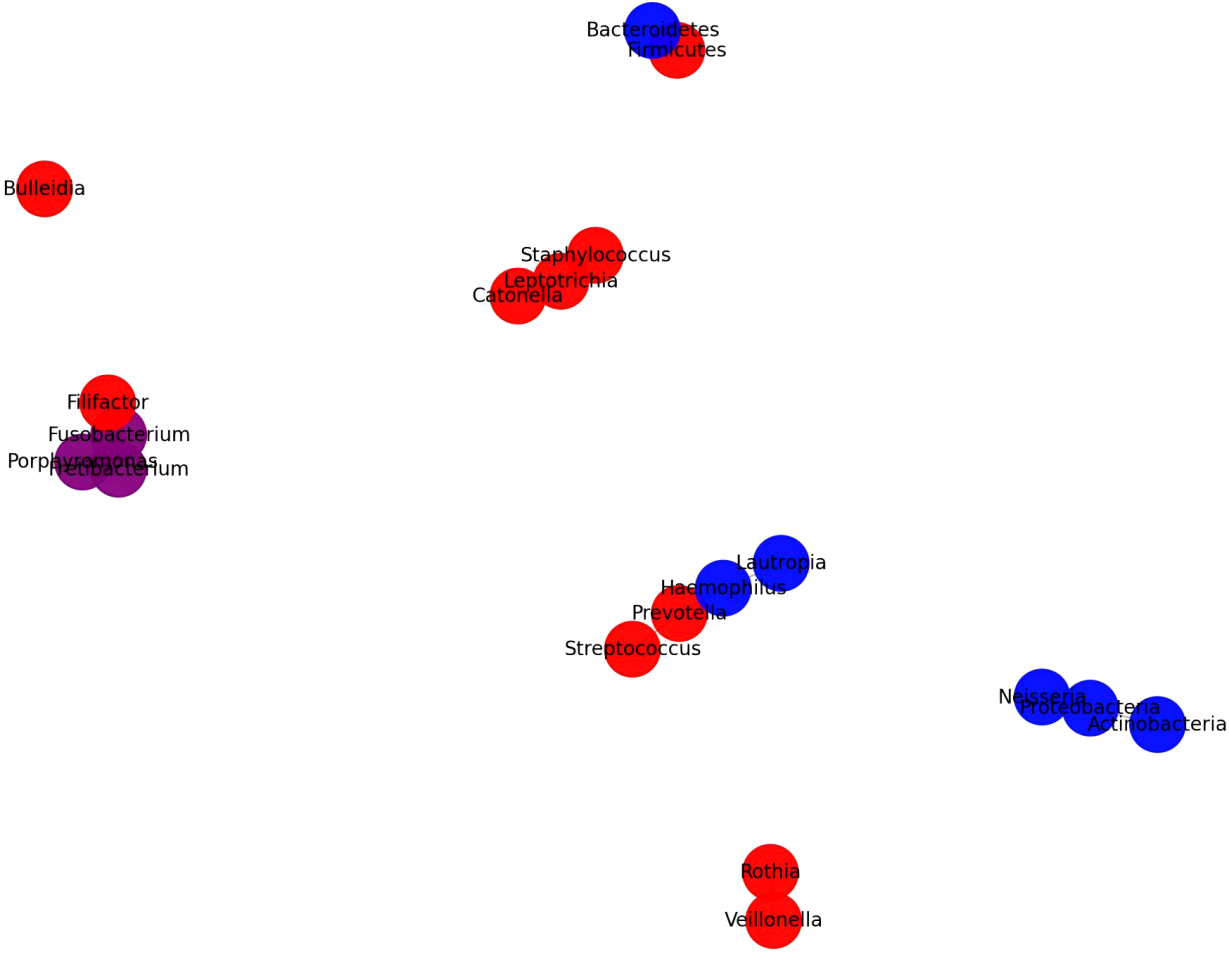
Interaction network of oral microbiota in pre-diabetes and T2DM contexts.

To further investigate the interactions within the oral microbiome specific to pre-diabetes and T2DM states, we constructed this interaction network using data from **Table 2**, following the same methodology applied to **Figure 1**. This network identifies distinct and overlapping microbial signatures within T2DM, non-T2DM, and periodontitis groups. By highlighting the relative abundances and co-occurrence patterns of specific taxa, **Figure 2** provides insights into unique microbiome alterations associated with disease progression in T2DM contexts.

The microbiome interaction network in **Figure 2** elucidates the differential abundance and interactions of key microbial taxa in T2DM, non-T2DM, and periodontitis groups, as derived from multiple studies. This diagram highlights a distinct microbial pattern associated with T2DM, underscoring both unique and overlapping taxa with non-T2DM and periodontitis groups. In T2DM patients, notable taxa like Streptococcus, Haemophilus, and Rothia are more abundant, reflecting a shift toward gram-positive bacteria that may play roles in inflammatory and metabolic disruptions linked to T2DM pathophysiology. Conversely, taxa such as Neisseria and Capnocytophaga are highlighted as more abundant in non-T2DM groups, suggesting that these taxa are linked to healthier oral microbiota profiles.

A significant overlap is observed between T2DM and periodontitis groups, with shared taxa such as Fusobacterium and Porphyromonas, which are associated with pro-inflammatory environments and periodontal disease. This overlap suggests that T2DM may exacerbate susceptibility to periodontitis through common microbial profiles that promote inflammation. Additionally, the interaction network reveals co-occurrence patterns among taxa; for instance, Veillonella and Prevotella show strong interactions within T2DM profiles, likely contributing to the short-chain fatty acid production associated with metabolic dysregulation in T2DM.

The network’s structure, with edges reflecting interaction strength, indicates that microbial dysbiosis in T2DM is both taxonomically distinct and functionally complex, potentially influencing both local oral health and systemic inflammatory states. The integration of taxa common to T2DM and periodontitis groups further underscores a bidirectional influence between these conditions, pointing to potential biomarkers and therapeutic targets that address both metabolic and periodontal health.

This visual summary reinforces the need for targeted microbiome-based diagnostics and interventions in managing T2DM and its complications, as the interlinked microbial community dynamics are integral to both metabolic and periodontal health.

## 6 Standardizing methods and integrating confounders: enhancing the reliability of oral microbiome studies in T2DM research

Although there is extensive data on oral microbiome-systemic disease links in current literature, the level of evidence remains unsatisfactory due to inconsistent study design between studies. To understand the influence of study design and confounding variables on the observed results, we evaluated current sequencing-based studies on oral microbiome-systemic disease links. The divergence between our results and past studies indicates the need for a unified study design for sample collection and the methods used for statistical analysis, when we to assess microbiome sequencing data from oral mucosal samples (19).

Confounding variables, encompassing both immutable characteristics like age, gender, and genetics, and adjustable factors such as lifestyle, oral health status, and other systemic conditions, play a pivotal role in shaping the oral microbiome landscape (40). These variables serve as critical determinants that may sway the variations observed within the oral microbiome, thereby influencing the interpretative differences across research findings (40). The nuanced impact of these confounding factors extends beyond mere coincidental associations, necessitating their deliberate integration and meticulous analysis within study designs. Their consideration is paramount, as it enhances the robustness and validity of conclusions drawn from oral microbiome studies, ensuring that interpretations are grounded in a comprehensive understanding of both intrinsic and extrinsic influences.

After analyzing our study of diabetic and ND individuals, including the full range of pre- and post-diabetic treatment phases, it’s clear there is no consensus on the results yet. Our investigation reveals dysbiosis within the oral microbiome may contribute to a bidirectional relationship between T2DM, periodontitis, and hyperglycemia. The differential enrichment of this microbiota may be related to the microenvironment changes in the oral cavity and the destruction of the host immune system in diabetic patients, suggesting that the characteristics displayed by the oral microbiota act as potential markers reflecting the disease state. However, the abundance of T2DM-related taxa varied in these studies, possibly because of the different sampling locations, patient statistics, and sequencing demographics (16, 19, 41).

Standardization of technical methods for microbiomics, alongside the standardization of big data algorithms and machine learning for bioinformatics that analyze the data, is critical for elucidating the compositional and functional differences in the oral microbiome in relation to T2DM (40, 42). High-throughput sequencing technologies, including 16S rDNA sequencing and whole-genome analysis, are pivotal in revealing microbial diversities. Nonetheless, the need for a comparative analysis stems from the inherent limitations associated with each 16S rDNA region. Variations in selecting hypervariable regions for sequencing underscore the importance of standardized methodologies to enable consistent interpretations across studies. A recent study comparing primers for 16S rRNA gene sequencing highlights the critical role of primer selection in oral microbiome research. The study evaluated six primer sets targeting different regions (V1-V2, V1-V3, V3-V4, V4-V5, V5-V7, and V6-V8), with V1-V2 primers showing the best performance for capturing original sequences and identifying taxa like Streptococcus (43). The findings emphasize that primer choice can significantly impact taxonomic resolution and consistency across studies. Using standardized primers, such as those targeting the V1-V2 region, can improve reproducibility and data comparability, which is especially important in T2DM-related microbiome research where precision in microbial identification is crucial (43).

Moreover, the choice and robustness of bioinformatics pipelines, essential for transforming 16S rRNA gene amplicon sequencing data into operational taxonomic units, significantly impact statistical test outcomes, thus affecting research comparability. These issues, coupled with challenges in data accessibility and the detailed description of methodologies, highlight the necessity for enhanced methodological clarity and comprehensive data deposition. Addressing these challenges by adopting reproducibility practices, such as multiple subsample analyses, is essential for deepening our understanding of the oral microbiome’s role in T2DM and promoting a unified framework for microbiome research. Artificial intelligence and its application in oral-systemic link prediction Advancements in high-throughput sequencing techniques have generated a vast amount of sequencing data, providing new insights into the oral-systemic link despite current setbacks in analysis and study design. The next step would be the prediction of disease states from the oral microbiome using AI tools such as ML and DL methods. For example, predictions using the Random Forest model had high accuracy rates, suggesting that the use of salivary bacteria for disease diagnosis has great potential and warrants future validation and optimization (34, 44).

These discrepancies highlight the need for standardized approaches in microbiomic research, particularly in the context of diabetes, where the consistency in sampling and analysis can significantly influence the interpretability and reliability of findings. By integrating these considerations, our investigation not only contributes to the broader understanding of the microbial underpinnings of T2DM but also emphasizes the necessity of a unified, standardized framework in microbiomic studies. This framework is essential not just for clarifying the role of the oral microbiome in diabetes but also for paving the way toward innovative diagnostic and therapeutic solutions. In this regard, the journey through the microbial landscapes of diabetes mirrors the broader challenges of defining and addressing metabolic health, advocating for a nuanced, multidisciplinary approach to unraveling the microbial dimensions of systemic diseases.

## 7 Discussion

In this section, we synthesize the findings from recent studies on the interaction between the oral microbiome, T2DM, and periodontitis, integrating results from our review to provide a comprehensive overview of their interrelated mechanisms.

### 7.1 Influence of T2DM on oral microbiota composition

The studies reviewed highlight how T2DM, especially in the presence of hyperglycemia, influences oral microbiota composition, increasing certain pathogenic taxa. Advanced sequencing methods, including 16S rRNA Gene Sequencing and Whole Metagenomic Shotgun Sequencing, reveal a prevalence of specific bacterial taxa, such as Fusobacterium nucleatum subsp. nucleatum, in diabetic populations. These findings support the role of diabetes-associated dysbiosis as a marker for T2DM progression and underscore the potential of microbial biomarkers for early detection and management.

### 7.2 Periodontitis and its bidirectional relationship with T2DM

The bidirectional relationship between periodontitis and T2DM was evident across studies. For instance, nonsurgical periodontal therapy improved both oral microbiota and glycemic control in T2DM patients. In addition, research on the microbial shifts induced by hypoglycemic treatments identified potential salivary biomarkers, supporting the interdependent nature of periodontal and systemic health. This highlights the need for an integrated therapeutic approach to address both oral and metabolic health.

The investigation into oral microbiota across pre-diabetes and T2DM stages uncovers a sophisticated interplay between elevated blood glucose and microbial dysbiosis in the oral cavity. This nuanced relationship is highlighted by significant findings, such as the distinct microbial enrichment of genera like Leptotrichia and Staphylococcus in individuals with higher glycemic levels (29).

### 7.3 Impacts of lifestyle and interventions on microbial dysbiosis

Our review reveals that the oral microbiome is not only impacted by T2DM but is also shaped by lifestyle factors and dietary interventions, such as adherence to a Mediterranean diet. These findings underscore the role of dietary and lifestyle interventions as promising adjuncts to conventional therapy, paving the way for holistic approaches to diabetes management.

### 7.4 Advancements in microbiomic research methodologies

The progression from traditional 16S rRNA sequencing to advanced multi-omics approaches, including metabolomics and lipidomics, has enhanced our understanding of microbial composition differences in T2DM patients. However, the lack of standardized methodologies across studies remains a limitation. Confounding factors, such as age, gender, and lifestyle, must be controlled, and consistent sampling and sequencing protocols are essential for reproducible results. Standardized methodologies will improve comparability and reliability, allowing for more accurate identification of disease-specific microbial signatures.

### 7.5 Future directions and the role of AI in microbiome research

The integration of artificial intelligence (AI) into microbiome research holds promise for advancing diagnostics and personalized treatments. By leveraging AI, predictive models could enhance our understanding of disease mechanisms, offering precision diagnostics based on microbiome data. Standardization in microbiome research will enable AI applications to better serve global health needs by identifying microbial biomarkers associated with systemic diseases like T2DM (45).

## 8 Conclusion

Our review highlights the complex interactions between oral microbiota, T2DM, and periodontitis. Through synthesizing data from advanced microbiomic techniques, it is evident that targeted interventions and lifestyle modifications play critical roles in managing these interconnected health issues. Moving forward, the development of standardized methodologies and integration of AI in microbiome research will be key to translating these findings into actionable insights for global health improvements.

In this context, the emphasis on methodological clarity and the integration of advanced bioinformatics tools, such as artificial intelligence, becomes a catalyst for not only enhancing our understanding of diseases like T2DM but also for facilitating access to innovative healthcare solutions across different regions and cultures.
